# Notch γ-Secretase Inhibitor Dibenzazepine Attenuates Angiotensin II-Induced Abdominal Aortic Aneurysm in ApoE Knockout Mice by Multiple Mechanisms

**DOI:** 10.1371/journal.pone.0083310

**Published:** 2013-12-16

**Authors:** Yue-Hong Zheng, Fang-Da Li, Cui Tian, Hua-Liang Ren, Jie Du, Hui-Hua Li

**Affiliations:** 1 Department of Vascular Surgery, Peking Union Medical Hospital, Beijing, China; 2 Department of Physiology and Physiopathology, Beijing AnZhen Hospital the Key Laboratory of Remodeling-Related Cardiovascular Diseases, Beijing Key Laboratory of Cardiovascular Diseases Related to Metabolic Disturbance, School of Basic Medical Sciences, Capital Medical University, Beijing, China; Beijing Institute of Heart, Lung and Blood vessel Diseases, Beijing, China; Medical University Innsbruck, Austria

## Abstract

Abdominal aortic aneurysm (AAA) is a life-threatening aortic disease in the elderly. Activation of *Notch*1 pathway plays a critical role in the development of AAA, but the underlying mechanisms remain poorly understood. In the present study, we explored the mechanisms by which *Notch*1 activation regulates angiotensin II (Ang II)-induced AAA formation and evaluated the therapeutic potential of a new *Notch* γ-secretase inhibitor, dibenzazepine (DBZ), for the treatment of AAA. Apolipoprotein E knockout (Apo E^−/−^) mice infused for 4 weeks with Ang II (1000 ng/kg/min, IP) using osmotic mini-pumps were received an intraperitoneal injection of either vehicle or 1 mg/kg/d DBZ. *Notch*1 signaling was activated in AAA tissue from both Ang II-infused Apo E^−/−^ mice and human undergoing AAA repair in vivo, with increased expression of Notch intracellular domain (NICD) and its target gene Hes1, and this effect was effectively blocked by DBZ. Moreover, infusion of Ang II markedly increased the incidence and severity of AAA in Apo E^−/−^ mice. In contrast, inhibition of *Notch* activation by DBZ prevented AAA formation in vivo. Furthermore, DBZ markedly prevented Ang II-stimulated accumulation of macrophages and CD4^+^ T cells, and ERK-mediated angiogenesis, simultaneously reversed Th2 response, in vivo. In conclusion, these findings provide new insight into the multiple mechanisms of *Notch* signaling involved in AAA formation and suggest that γ-secretase inhibitor DBZ might be a novel therapeutic drug for treating AAAS.

## Introduction

Abdominal aortic aneurysm (AAA) is a leading cause of sudden death in aging (> 65 years) men. The exact molecular mechanisms for the initiation, progression and rupture of AAAs has not been fully defined [1]. Pathologically, AAA tissues from both human and animal models are characterized by vascular remodeling, immune responses, degradation of extracellular matrix (elastin and collagen), vascular cell apoptosis, and neovascularization of the media and adventitia [2]. Numerous mechanisms are known to contribute to aortic dilatation formation, but the unique pathways driving this process are incompletely understood. Recently, several signaling pathways, including AMPK, ERK and *Notch*1, have been proved to play a critical role in AAA formation [1,2]. 

The *Notch* family, including *Notch* 1-4, acts as receptors and is essential for cellular growth, differentiation, apoptosis and vessel formation. Upon ligand binding, the intracellular domain of *Notch* (NICD) is released by proteolytic cleavage processes via ADAM metalloproteases and γ-secretase, respectively, leading to its nuclear translocation and induction of target genes such as Hairy enhancer of split (Hes) [3] . Genetic studies of mice have demonstrated an essential role of *Notch* signaling in vascular remodeling [4,5]. Abnormal activation of *Notch* signaling has been implicated in the pathogenesis of various diseases, such as atherosclerosis, pulmonary arterial hypertension, and large-vessel vasculitis [6-8]. Recently, Hans et al demonstrate that *Notch*1 activation promotes AAA formation through macrophage-mediated inflammation [3]. However, the exact mechanisms by which *Notch* signaling contributes to the development of AAA remain to be explored. 

 Over the past decades, small molecule inhibitors for γ-secretase activity have been actively investigated for their potential to block the generation of Aβ-peptide that is associated with Alzheimer’s disease [9]. Because γ-secretase inhibitors (GSIs) are also able to effectively inhibit *Notch* receptor signaling, several forms of γ-secretase inhibitors, including N-[N-(3,5-difluorophenacetyl)-l-alanyl]-Sphenylglycine *t*-butyl ester (DAPT), compound E, and IL-X (cbz-IL-CHO) MRK-003 and dibenzazepine (DBZ), have been tested for the treatment of tumor and cardiovascular diseases [6,7,10]. Particularly, γ-secretase inhibitors have been shown to have both anti-inflammatory and anti-proliferative properties [3,8,10,11]. These include the inhibition of macrophage and T cell infiltration, M1/M2 transition and cytokine expression [3,8]. Furthermore, GSIs have been reported to inhibit angiogenesis for VEGF- and Ang II-stimulated new blood vessel formation [12] . Both vascular inflammation and angiogenesis are involved in AAA formation, suggesting that GSIs might prevent Ang II-stimulated AAA formation in Apo E^−/−^ mice.

 In the present study, we extended the previous results of *Notch* pathway involved in AAA formation and demonstrated that in addition to enhancing macrophage-mediated inflammation, *Notch* activation also promoted the accumulation of CD4^+^ T cells, Th2 differentiation and ERK-mediated angiogenesis by detecting the AAA tissues from human and mouse model. In contrast, the γ-secretase inhibitor, DBZ, markedly inhibited *Notch* activation-mediated effects resulting in decrease in both the extent and severity of Ang II-stimulated aneurysm. Thus, these results suggest that *Notch* pathway plays a critical role in the development of AAA via multiple mechanisms. The γ-secretase inhibitor DBZ might be a new therapeutic drug for the treatment of AAA disease.

## Results

### *Notch* signaling is activated in the abdominal aorta from human AAA tissue or Ang II-infused Apo E^-/-^ mice and is inhibited by γ-secretase inhibitor

**Figure 1:**
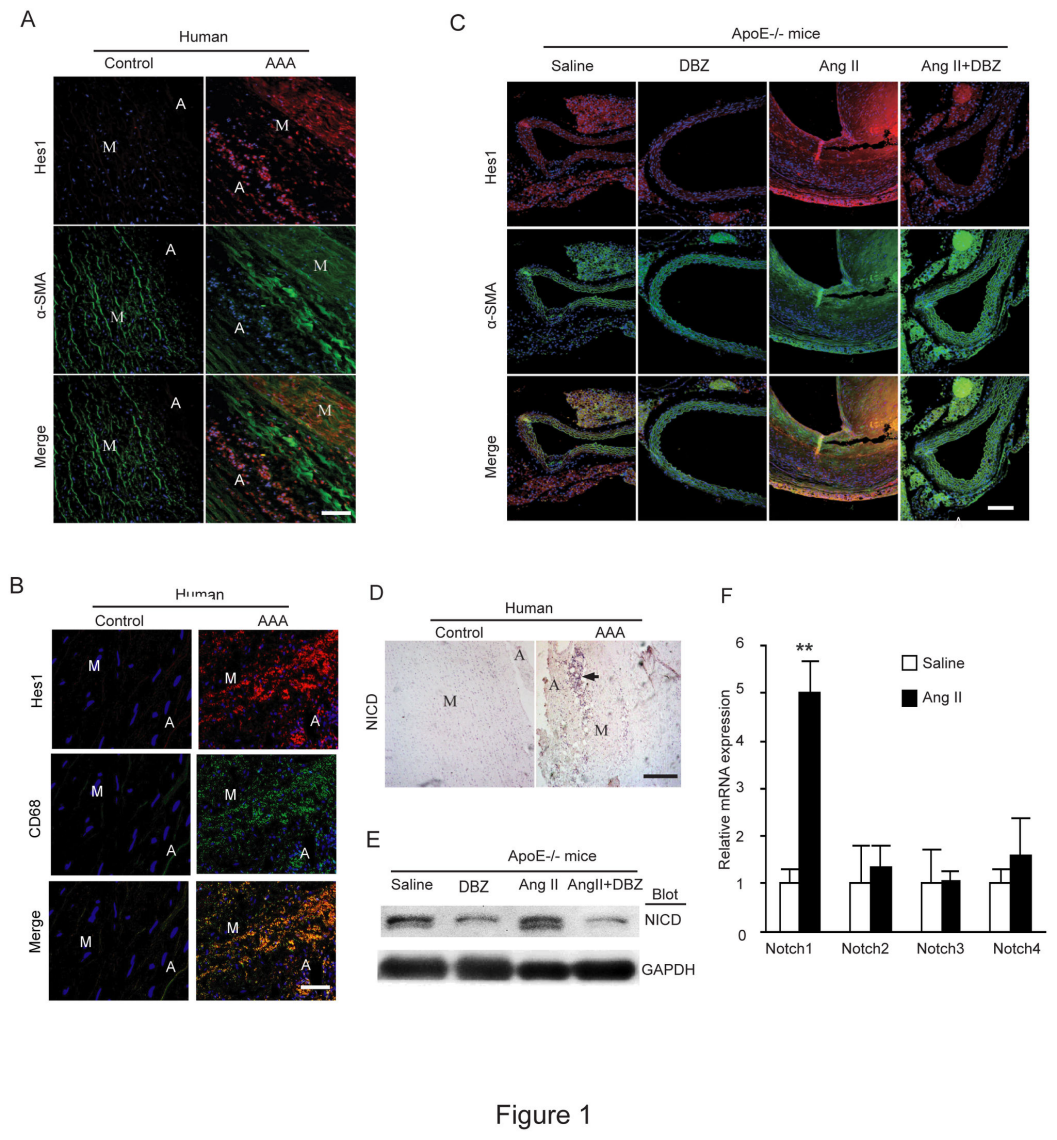
*Notch* signaling is activated in the abdominal aorta of human AAA tissue or Ang II-infused apo E^-/-^ mice and γ-secretase inhibitor inhibits this activation. (**A**) The expression of Hes1 (red) and α-smooth muscle-actin (α-SMA) (green) was detected by double immunostaining in human AAA tissues. Nuclei are counterstained with DAPI (blue). (**B**) The expression of Hes1 (red) and CD68 (green) was detected as in A. (**C**) The expression of Hes1 (red) and α-smooth muscle-actin (α-SMA) (green) was detected by double immunostaining in the aneurysmal abdominal aorta (AAA) tissue from Apo E^-/-^ mice at week 4 of angiotensin II (Ang II) infusion (n=3 per group). (**D**) The expression of Notch1 intracellular domain (NICD) was examined by immunohistochemistry in human AAA tissue. (**E**) The expression of NICD was examined by Western blot analysis in AAA tissue from Apo E^-/-^ mice (n=3) at week 4 of angiotensin II (Ang II) infusion (n=3 per group). (**F**) The expression of Notch 1-4 was analyzed by qPCR analysis in AAA tissue from Apo E^-/-^ mice. GAPDH was used as an internal control. Scale bars: 50 μm. Data expressed as mean±SEM (n=3). **P < 0.01, vs. saline group. M: media; A: adventitia.

To investigate the role of *Notch* signaling in the AAA formation, we first examined the expression of NICD (the active form of *Notch*1) and its downstream effector Hes1 in the abdominal aorta of human AAA tissue or Ang II-infused Apo E^-/-^ mice. Immunofluorescent staining was performed in human AAA tissue with antibodies against Hes1 (a downstream target genes of NICD) and α-smooth muscle-actin (α-SMA) (a marker for smooth muscle cells). As shown in Figure 1A, the expression of Hes1 (red) in the adventitia and media and α-SMA (green) in the media was markedly increased in the human AAA tissue compared with age-matched control. Interestingly, Hes1 staining showed no overlapp with α-SMA, indicating that Hest1 was not mainly expressed in smooth muscle cells (SMCs). Similar results were observed in Ang II-infused Apo E^-/-^ mice (Figure 1C). Furthermore, Hes1 staining was co-localized with CD68 (a marker for human macrophages) in the adventitia and media of human AAA tissue (Figure 1B), suggesting that Hes1 was mainly expressed in the infiltrated macrophages. Immunohistochemistry (Figure 1D) and Western blot analysis (Figure 1E) further demonstrated that the expression of NICD protein was significant higher in the aneurysmal aorta of human AAA tissue and Ang II-treated Apo E^-/-^ mice than that in control or saline-treated Apo E^-/-^ mice. Finally, qPCR analysis confirmed that *Notch*1 receptor, but not others, was significantly up-regulated in Ang II-induced AAA mice compared with control mice (Figure 1E). Together, activation of the *Notch*1 pathway is observed in both mouse and human models of AAA and may play a role in AAA formation. 

To confirm that *Notch* γ-secretase inhibitor DBZ was efficient in disrupting *Notch* signaling, the expression of NICD and Hes1 was assessed in Ang II-infused aorta of mice by immunostaining or Western blot analysis. DBZ treatment significantly abrogated the expression of NICD and Hes1 in the aortic artery compared to non-treated group after saline or Ang II infusion (Figure 1C and E), indicating that the DBZ treatment effectively inhibits activation of *Notch* signaling in Apo E^-/-^ mice. 

### DBZ inhibits the formation of aneurysms in Ang II-infused Apo E^-/-^ Mice

**Figure 2:**
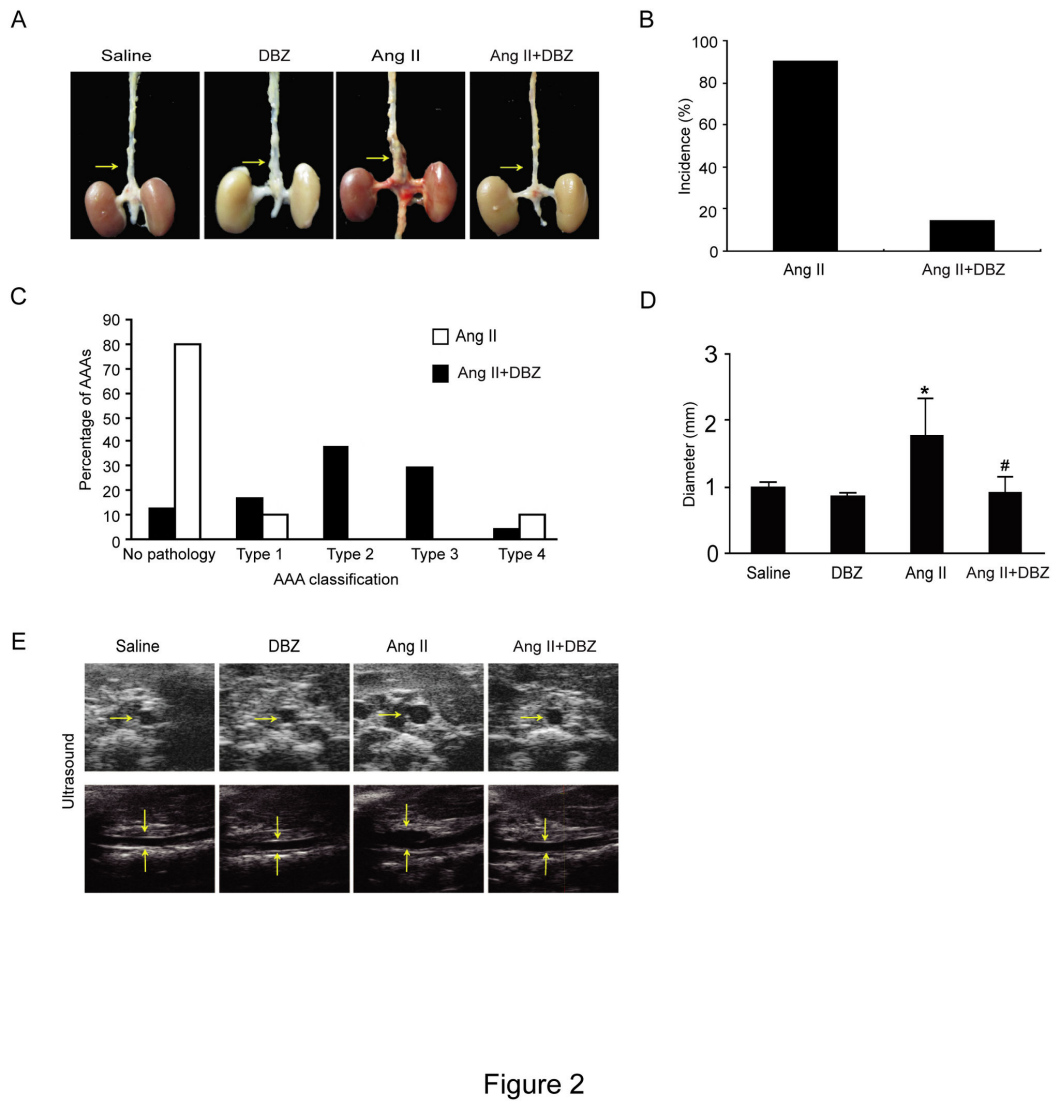
DBZ injection reduces the incidence and severity of abdominal aortic aneurysms in Ang II –infused apoE^-/-^ mice. Wild-type (WT) mice were injected with γ-secretase inhibitor DBZ (1 mg/kg/day, IP) or saline every day from 1 day before Ang II infusion (1000 ng/min/kg). (**A**) Representative aortas. Arrows indicate aortic aneurysms. (**B**) The incidence of AAA formation in mice infused with Ang II (n=24) or Ang II+DBZ (n=19). (**C**) Classification of aortic aneurysms in mice infused with Ang II or Ang II+ DBZ as described by Daugherty. (**D**) Aortic diameter. (**E**) Representative images by high-frequency ultrasound (ultrasound) of abdominal aortas. Transverse (top) and longitudinal (bottom) images were taken at the level of the suprarenal aorta. Data expressed as mean±SEM. *P < 0.05 vs control, ^#^ *P* < 0.05 vs Ang II.

To determine the effect of *Notch* signaling inhibitor DBZ on Ang II-induced AAA formation, Ang II-infused Apo E^-/-^ mice were treated subcutaneously with DBZ (1 mg/kg/d) or vehicle for 4 weeks. No aortic aneurysms were present in saline-infused control mice. Ang II infusion resulted in a dramatic increase in the incidence of AAA formation (87.5%, 21/24), and this effect of Ang II was markedly lower in Apo E^-/-^ mice treated with DBZ (15.8%, 3/19) (Figure 2A and B). Furthermore, based on the classification system of Daugherty et al [13], the aneurysms in the DBZ-treated group were less severe than those in the Ang II alone group. 70.8% (17/24) of mice treated with Ang II alone developed either Type II, III or IV aneurysms, while only 5.3 % (1/19) of Mice treated with Ang II+DBZ did (Figure 2C). Finally, the average suprarenal aortic diameter of Apo E^-/-^ mice treated with Ang II alone was larger than that in the saline control group; In contrast, this effect of Ang II was markedly attenuated in Apo E^-/-^ mice treated with Ang II+DBZ (Figure 2D). These results were further confirmed by high-frequency ultrasound (Figure 2E). Thus, inhibition of *Notch* signaling by DBZ markedly reduced the incidence and severity of Ang II-induced aneurysm formation.

### DBZ attenuates *Notch* signaling-mediated remodeling of the aortic wall in Ang II-infused mice.

**Figure 3:**
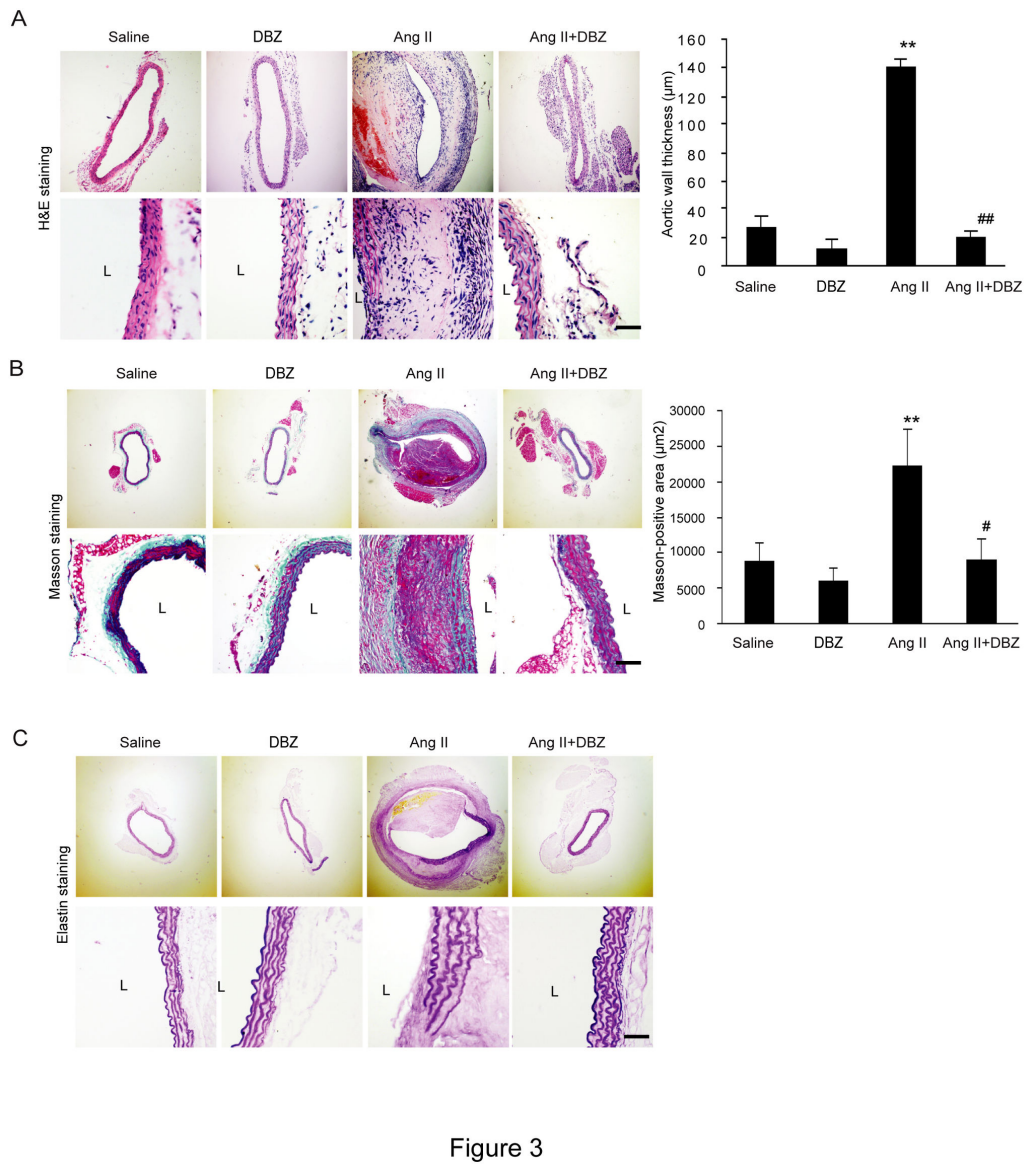
DBZ inhibits remodeling of the aortic wall in Ang II-infused mice. (**A**-**B**) The representation of H&E, Masson and elastin staining in abdominal aorta from four groups (left). Bar graph show the quantification of aortic wall thickness and the collagen deposition area (right; n=3 per group). (**C**) The representation of elastin staining in abdominal aorta from four groups. Magnification: x100. Bar: 50 μm. Data expressed as mean±SEM. **P<0.01 vs. sham group; ^#^ *P*<0.05 vs. Ang II group. L: lumen.

It has been known that extensive remodeling of the artery wall is a hallmark of aneurysm formation and progression in humans and Ang II-induced animal models [14,15]. Therefore, we determine the effect of *Notch* signaling on the remodeling of the suprarenal artery wall in Apo E^-/-^ mice using H&E, Masson trichome and VVG staining. As shown in Figure 3A-C, Ang II injection resulted in a thickening of the abdominal aortic wall, disruption of the media with thrombus formation, increase of the collagen deposition and breaks of the elastic fibers. However, these histological alterations were markedly suppressed in Apo E^-/-^ mice treated with Ang II+DBZ (Figure 3A-C). There was no significant difference in the alterations of the aortic wall remodeling between groups after saline infusion (Figure 3A-C).

### DBZ inhibits macrophage-mediated inflammation of the aortic wall in Ang II-infused mice

**Figure 4:**
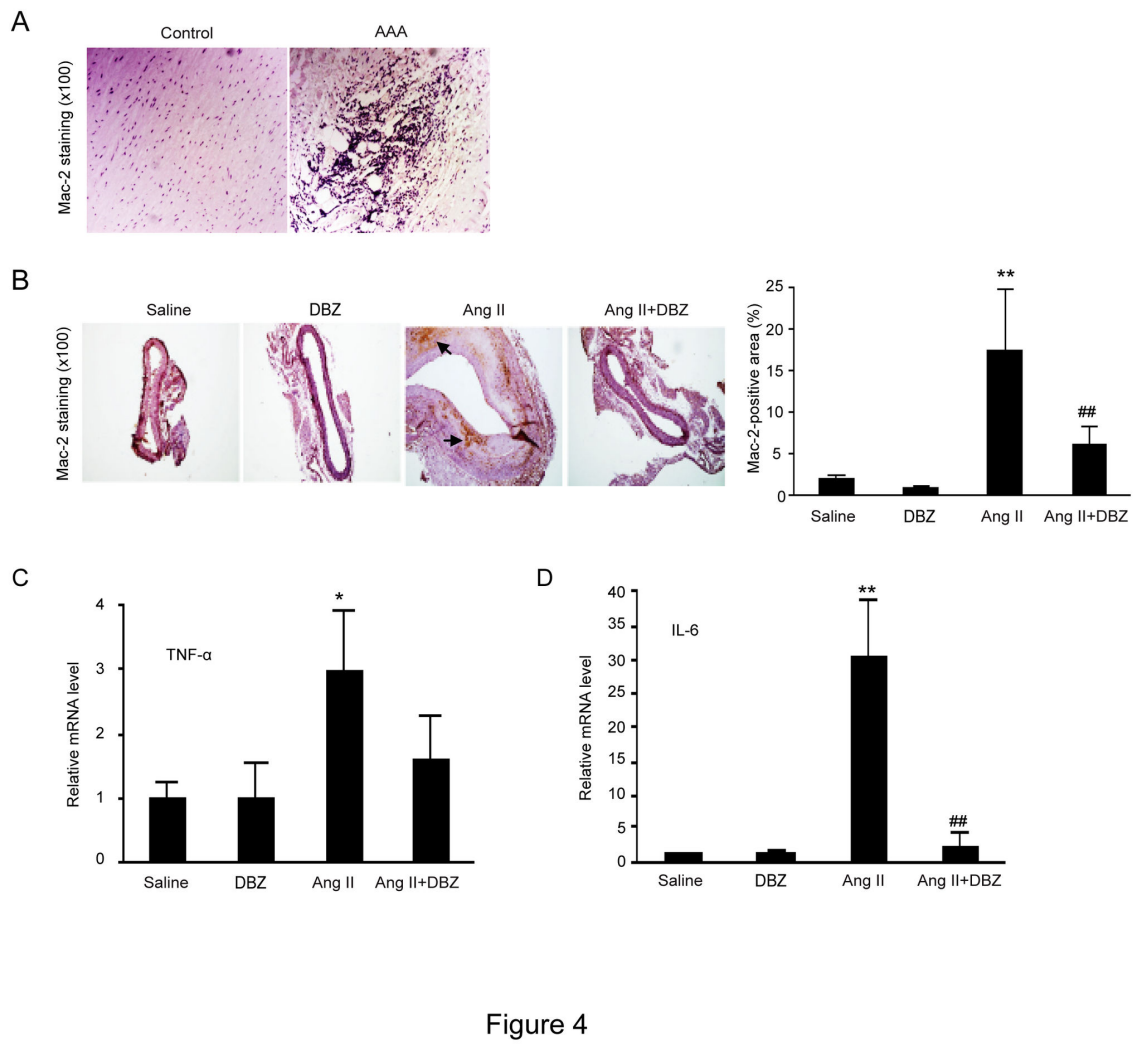
DBZ inhibits the accumulation of macrophages and the expression of inflammatory cytokines in the AAAs. (**A**) The representation of immunohistochemical staining for Mac-2 in abdominal aorta from human abdominal aortic aneurysms (AAA) and control. (**B**) The representation of immunohistochemical staining for Mac-2 in abdominal aorta from four groups (left). Bar graph show the percentage of Mac-2 positive cell areas (right; n=3 per group). Bar: 50 μm. (**C**) qPCR analysis for the mRNA expression of TNF-α and IL-6 in abdominal aorta. GAPDH was used as an internal control. Data expressed as mean±SEM. *P<0.05, **P<0.01 vs. sham group; #P<0.05, ##P<0.01 vs. Ang II group.

Since macrophage infiltration occurs during early and later stages of AAA development [16]. We then determine macrophage-mediated inflammation in the aortic wall of Ang II-infused mice by immunohistochemstry. There was significant infiltration of Mac-2-positive macrophages in the aortic aneurysms from both human patients (Figure 4A) and the Ang II-infused Apo E^-/-^ mice (Figure 4B). The expression of proinflammatory cytokines including TNF-α and IL-6 was markedly increased in the aneurysmal aorta of Ang II-treated Apo E^-/-^ mice (Figure 4C). However, these effects were markedly suppressed in Apo E^-/-^ mice treated with Ang II+DBZ (Figure 4B and C). These findings were in consistent with Hans’ work published recently [3,7] . 

### DBZ decreases proportion of CD4^+^ T lymphocytes in serum and in the aortic wall of Ang II-infused mice

**Figure 5:**
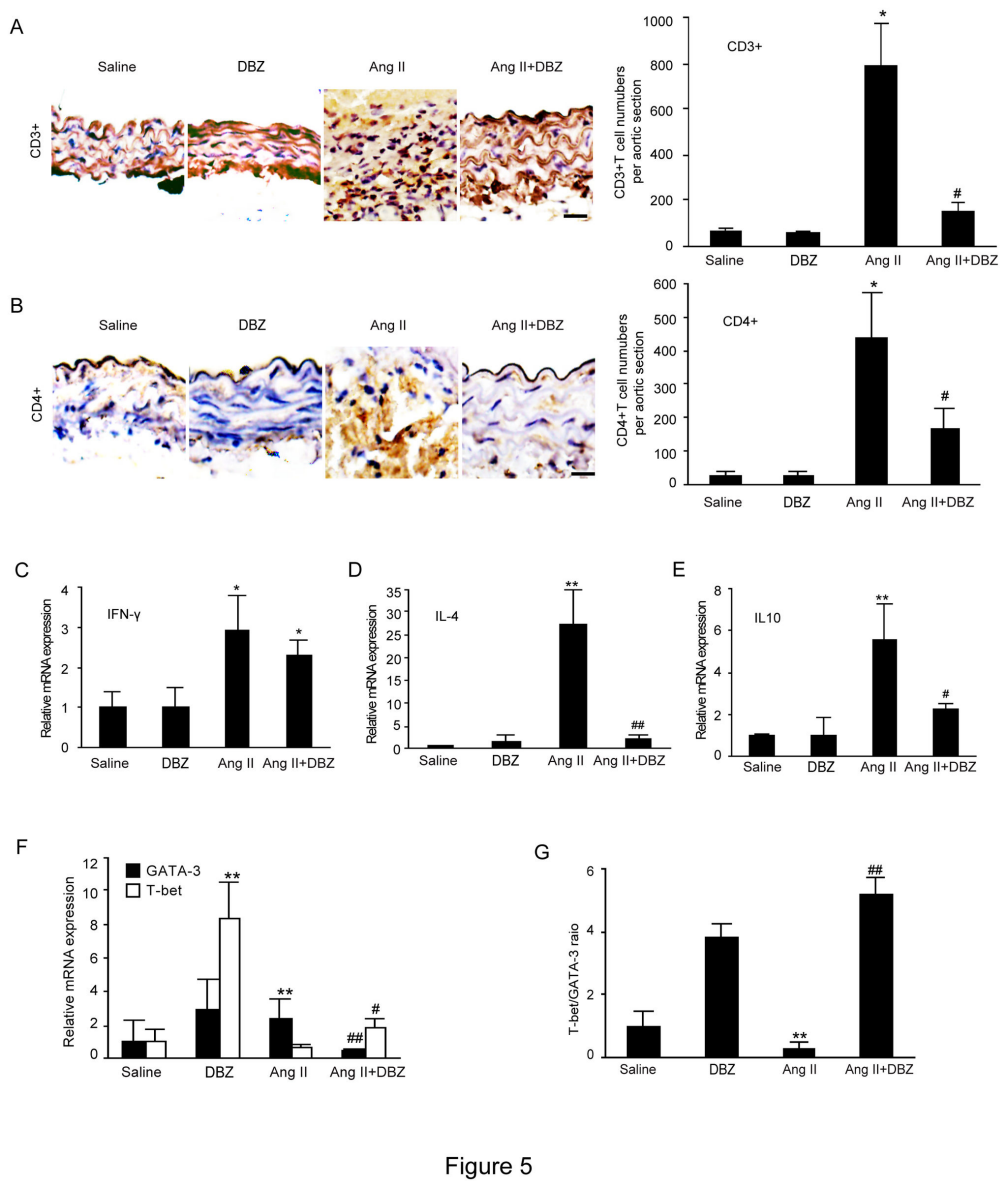
DBZ inhibits the accumulation of CD4^+^ T cells and Th2 differentiation in the AAAs. (**A**, **B**) The representation of immunohistochemical staining for CD3^+^ and CD4^+^ in abdominal aorta from four groups (left). Bar graphs show the percentage of CD3^+^ and CD4^+^ positive cell areas (right; n=3 per group). Bar: 50 μm. (**C**-**E**) qPCR analysis for the mRNA expression of IFN-γ, IL-4 and IL-10 in abdominal aorta from four groups (n=4 per group). (**F**) qPCR analysis for the mRNA expression of T-bet and GATA3 in abdominal aorta. (**G**) Bar graph shows the ratio of T-bet/GATA3 in E (n=4 per group). GAPDH was used as an internal control. Data expressed as mean±SEM. *P<0.05, **P<0.01 vs. sham group; ^#^ *P*<0.05, ^##^ *P*<0.01 vs. Ang II group.

T lymphocytes have been known to play a critical role in inflammatory response. To confirm the effect of *Notch* signaling on the accumulation of T cells, we first examined the total CD3^+^ T cells and proportions of CD4^+^ and CD8^+^ T cells in the serum of mice by flow cytometry. Total CD3^+^ T cells and the proportions of CD4^+^ cells were markedly elevated in the Ang II-treated group versus saline-treated control group (15.3% vs.10.2%, *P* < 0.05), whereas CD8^+^ T cells were similar between two groups (13.8% vs. 12.5%, *P* > 0.05). Following DBZ infusion, the proportions of CD4^+^ T cells, but not CD8^+^ T cells were significantly diminished compared to Ang II/Sham-treated group (3.5% vs. 15.3%, *P* < 0.05), indicating that peripheral CD4^+^ cells may play a key role in AAA formation. Moreover, immunohistochemistry of aortic wall further confirmed that CD3^+^ and CD4^+^ T cells were significantly increased in the AAAs of Ang II-treated Apo E^-/-^ mice compared with saline-infused Apo E^-/-^ mice. In contrast, DBZ treatment markedly attenuated these effects (Figure 5A-B). There was no significant difference in the infiltration of CD3^+^ and CD4^+^ T cells in the AAAs between groups after saline infusion (Figure 5A-B).

### DBZ predominantly inhibits Th 2 phenotype of the aortic wall in Ang II-infused mice

It is reported that *Notch* signaling participates in Th cell differentiation [17], and Th2 subset has a role in aneurysm formation [18]. To determine the impact of *Notch*1 signaling on the differentiation of CD4^+^ T cells in AAAs, we detected the mRNA expression of the cytokines that mark these T helper subsets (IFN-γ as a marker for Th1; IL-4 and IL-10 as markers for the Th2) by qPCR analysis. The mRNA expressions of IFN-γ, IL-4 and IL-10 were significantly increased in the aortic aneurysms of Ang II-treated group compared with saline-treated controls (Figure 5 C-E). Th1 cell expression of IFN-γ was similar between two groups (Figure 5C). In contrast, Th2 cell expression of IL-4 and IL-10 was markedly diminished in Ang II/DBZ-treated group (Figure 5D and E), indicating that the inhibition of *Notch* signaling attenuated infiltrating CD4^+^ T cells differentiated into Th2 phenotype. 

Since differentiation of CD4^+^ T lymphocytes into Th1 or Th2 cells is reciprocally regulated by the T-bet and GATA-3 transcription factors, respectively [19,20]. Increased T-bet/GATA-3 expression ratios favors induction of the Th1 response [21]. We then quantitated the levels of T-bet and GATA-3 mRNA expression in the aortic artery by qPCR analysis. Ang II infusion significantly decreased the T-bet mRNA level but increased GATA-3 expression in the aortic aneurysms compared to saline-treated controls, and these effects were markedly reversed in Ang II/DBZ-treated group (Figure 5F). The ratio of T-bet/GATA-3 was also significantly lower in Ang II/DBZ-treated aortic aneurysms compared to Ang II-treated group (Figure 5G). Thus, in the development of AAAs, inhibition of *Notch* signaling of T lymphocytes by DBZ favors Th2 differentiation leading to AAAs formation.

### DBZ inhibits Ang II–induced MMP-2, MMP-9 and MCP-1 expression in Ang II-Induced AAA tissue

**Figure 6:**
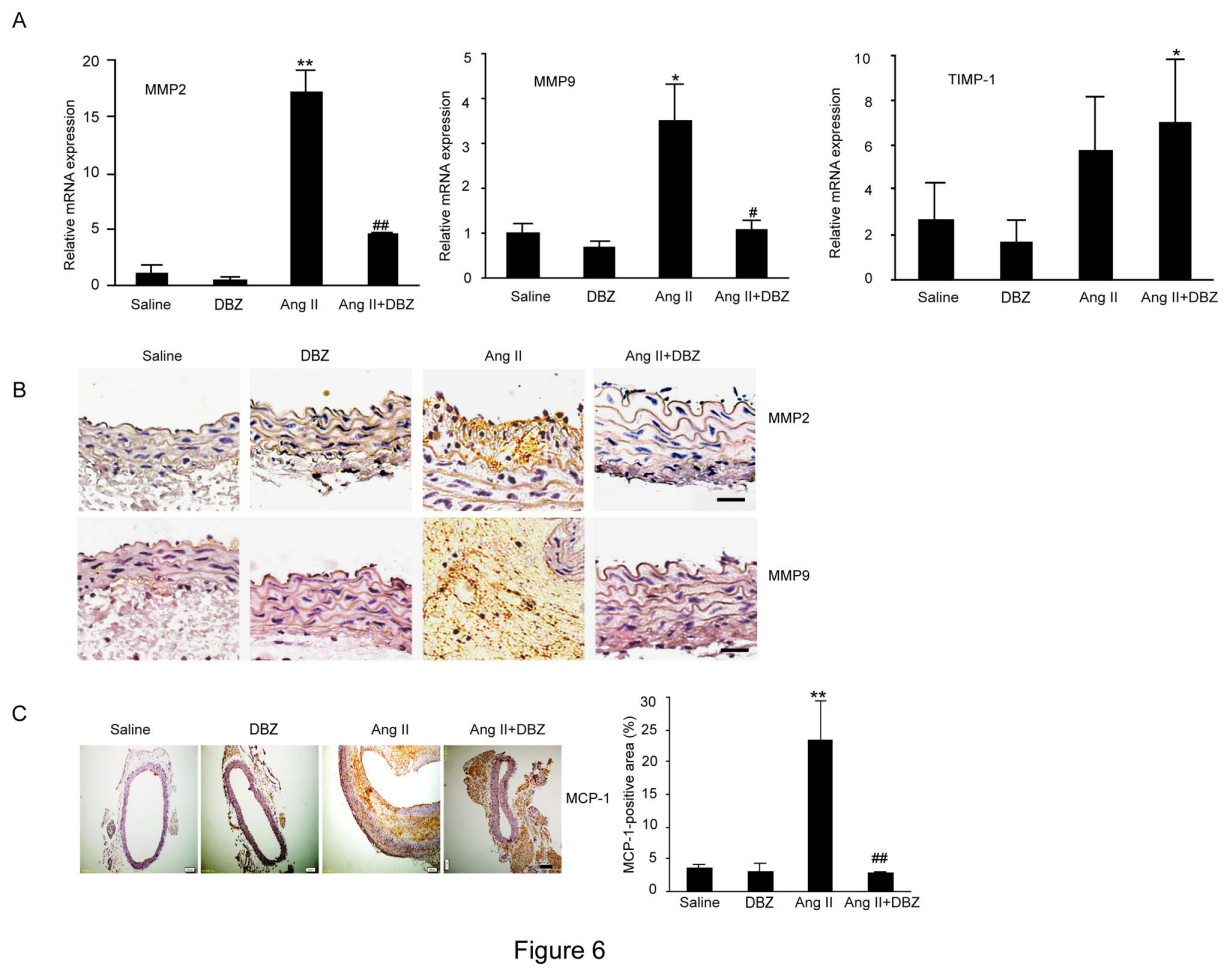
DBZ inhibits the expression of MMP-2, MMP-9 and MCP-1 in the AAAs. (**A**) The qPCR analysis for the mRNA expression of MMP-2, MMP-9 and TIMP in abdominal aorta from four groups (n=4 per group). GAPDH was used as an internal control. (**B**) The representation of immunohistochemical staining for MMP-2 and MMP-9 in abdominal aorta from four groups (n=3 per group). Bar: 50 μm. (**C**) The representation of immunohistochemical staining for MCP-1 in abdominal aorta (left). Bar graph shows the percentage of MCP-1 positive cell areas (right; n=3 per group). Bar: 50 μm. Data expressed as mean±SEM. *P<0.05, **P<0.01 vs. sham group; ^#^ *P*<0.05, ^##^ *P*<0.01 vs. Ang II group.

Ang II has been reported to stimulate the expression of MMPs during AAA formation. *Notch* activation is known to induce MMP-2 and MMP-9 secretion [22]. We then analyzed the expression of aortic MMP-2, MMP-9 and TIMP from saline- and Ang II–infused Apo E^-/-^ mice by qPCR. We found that the expression of MMP-2 and MMP-9 mRNA was significantly increased at 4 weeks after Ang II infusion in Apo E^-/-^ mice. In contrast, this increase was markedly suppressed in DBZ-treated Apo E^-/-^ mice (Figure 6A, left and middle). Otherwise, DBZ did not significantly influenced TIMP expression under saline or Ang II stimulation (Figure 6A, right). Furthermore, immunohistochemical staining also demonstrated that DBZ treatment markedly inhibited Ang II-induced expression of MMP-2 and MMP-9 in aortic aneurysmal tissue compared with control (Figure 6B). In addition, we also detected MCP-1 expression by immunohistochemistry, which is the primary chemoattractant for monocyte transendothelial migration and major cytokine during the development of AAAs. Ang II infusion in Apo E^-/-^ mice markedly up-regulated MCP-1 expression compared with saline-infused control Apo E^-/-^ mice, and this effect was attenuated by DBZ injection (Figure 6C). These results indicate that DBZ reduced the extent and severity of Ang II-stimulated AAA formation in association with a decrease in activity of MMPs. 

### DBZ decreases Ang II-induced neovascularization of aneurysmal tissue and activation of ERK signaling

**Figure 7:**
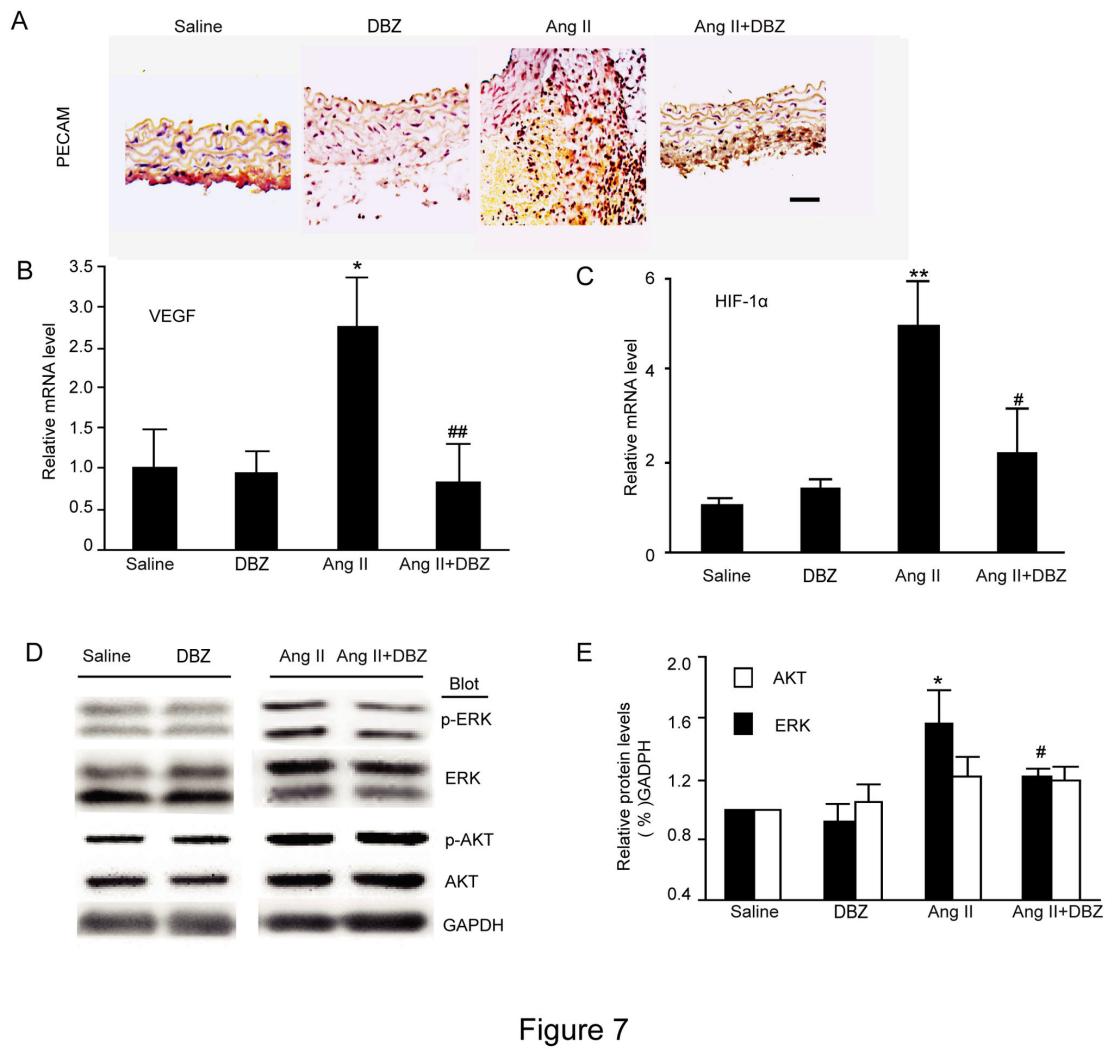
DBZ attenuates Ang II-induced neovascularization in aortic tissue. (**A**) The representation of immunohistochemical staining for PECAM in abdominal aorta from four groups (n=3 per group). Bar: 50 μm. (**B**-**C**) qPCR analysis for the mRNA expression of VEGF and HIF-1α in abdominal aorta from four groups (n=4 per group). GAPDH was used as an internal control. (**D**) Western Blot analysis for the protein levels of total and phosphorylated ERK1/2 and AKT in abdominal aorta from four groups. (**E**) Bar graph shows the quantification relative to GAPDH level (right, n=3 per group). Data expressed as mean±SEM. *P<0.05, **P<0.01 vs. sham group; ^#^ *P*<0.05, ^##^ *P*<0.01 vs. Ang II group.

 Given the activation of *Notch* signaling induces angiogenesis through increasing the expression of HIF-1α and its target gene VEGF [4,5,12] ,and mural angiogenesis is a prominent pathologic feature in AAAs [23,24], we then performed immunohistochemical staining of aortic aneurysmal tissue with PECAM antibody. The numbers of capillary (determined by PECAM-staining) in AAAs of Ang II-treated Apo E^-/-^ mice were markedly higher compared to aortas of control Apo E^-/-^ mice (Figure 7A). Moreover, qPCR analysis revealed that the mRNA expression of VEGF and HIF-1α were significantly up-regulated in the AAAs of Ang II-treated group compared to saline-treated controls. In contrast, DBZ treatment markedly attenuated these effects (Figure 7B and C). 

 Given the role of *Notch* in regulation of ERK and AKT activation, which induces MMP secretion and angiogenesis [4,5], we then determined the effect of DBZ on ERK and AKT activation in homogenates of aneurysmal tissue. The relative levels of p-ERK and p-AKT in extracts of AAAs from Ang II-treated Apo E^-/-^ mice were markedly higher compared to aortas of control Apo E^-/-^ mice (Figure 7D and E). In contrast, DBZ treatment markedly inhibited ERK phosphorylation. However, DBZ treatment had no effect on AKT phosphorylation (Figure 7D and E)

## Discussion

In the present study, we demonstrate that Ang II-mediated activation of *Notch*1 signaling significantly enhanced the AAA formation in ApoE^−/−^ mice, whereas *Notch* γ-secretase inhibitor DBZ markedly attenuated this effect. These data expands on previous results, in addition to inhibiting macrophage infiltration, inhibition of *Notch* signaling by DBZ also reduced the accumulation of CD4^+^ T cells, Th2 differentiation, expression of MMP-2 and MMP-9, and ERK-mediated angiogenesis. Thus, our data provide the novel in vivo evidence for the critical role of *Notch* inhibition in prevention of AAA in ApoE^−/−^ mice after Ang II infusion, and suggest that γ-secretase DBZ might be a new therapeutic drug for the treatment and prevention of AAAs. 

*Notch* signaling pathway plays a critical role in the pathogenesis of a variety of inflammatory diseases and vascular remodeling [25]. Pharmacological γ-secretase inhibitors (GSIs) effectively prevent activation of all *Notch* receptors by inhibiting this final enzymatic cleavage [26]. GSIs have been used to treat lymphoblastic leukemia and melanoma, reduce atherosclerotic lesion formation [6], reverse pulmonary hypertension [7] and prevent progression of system sclerosis [27]. Particularly, GSI treatment reduces allergic pulmonary inflammation by decreasing Th2 cytokine production and concomitantly increasing Th1 cytokine secretion [10]. Recently, *Notch* activation has been reported to promote AAA formation through macrophage-mediated inflammation [3,28]. However, the molecular mechanisms by which *Notch*1 promotes AAA formation have not been fully illustrated. Consistent with previous results [3], activation of *Notch*1 by Ang II infusion markedly increased macrophage infiltration and expression of cytokines (Figure 4). In addition, we also demonstrated, for the first time, that Ang II-mediated activation of *Notch*1 signaling enhanced accumulation of CD4^+^ T cells, Th2 differentiation and cytokine production, and ERK-mediated neovascularization, leading to AAA formation in Apo E^−/−^ mice. In contrast, γ-secretase inhibitor DBZ markedly attenuated these effects (Figure 5 and 7). Moreover, although Ang II increased blood pressure in Apo E^-/-^ mice, we demonstrated that the attenuation of Ang II-stimulated AAA formation by DBZ was independent of any effect on the Ang II-trigged hypertension in these mice (data not shown). The conclusion was in consistent with previous observations. For example, norepinephrine infusion induced high blood pressure in Apo E^-/-^ mice but did not induce aneurysm formation [22]. Treatment with antihypertenisve drug hydralazine did not affect the incidence of AAA formation in Ang II-treated Apo E^-/-^ mice [28] . Furthermore, our data indicated that DBZ had no effect on the lipid profile in Apo E^-/-^ mice after Ang II infusion (data not shown). Together, these results demonstrate that *Notch*1 signaling pathway plays an important role in the development of AAA via multiple mechanisms.

The pathologic characteristics of AAA classically consist of inflammatory infiltrates with elastin and collagen (types I and III) degradation, resulting in vessel wall expansion and potentially rupture [29]. However, the precise molecular mechanisms remain unclear. Recently, AAAs is considered as a chronic inflammatory disease. Proinflammatory cells such as macrophages and T cells may play a critical role in the pathogenesis of the disease by releasing cytokines, chemokines and adhesion molecules [16]. Activation of macrophages causes activation of MMPs, leading to both the degradation of elastin and collagen, and smooth muscle cell apoptosis, indicating that macrophages play pivotal roles in AAA formation [16]. Macrophages as critical regulators of inflammation can differentiate into classically activated M1 or alternatively activated M2 macrophages. *Notch* signaling plays a critical role in the determination of M1 versus M2 polarization of macrophages, and compromised *Notch* pathway activation will lead to the M2-like anti-tumor-associated macrophages (TAM) [22]. In Notch1^+/−^Apo E^−/−^ mice, a significant reduction of the expression of chemokines and cytokines including MCP-1, IL-6, Cxcl10, VEGF, iNOS, ICAM-1, VCAM-1 was observed compared with Notch1^+/+^Apo E^−/−^ mice [3,28]. Moreover, LPS/interferon-γ induced expression of IL-6, IL-12, TNF-α, and iNOS genes in the BMDM of Apo E^−/−^ mice, whereas no change was observed in Notch1^−/+^ Apo E^−/−^ mice, suggesting that *Notch1*-deficient macrophages were polarized toward the M2 phenotype [3,28]. Consistent with previous results, our data also demonstrated that Ang II markedly increased macrophage accumulation and expression of macrophage cytokine IL-6, IL-1 and TNF-α in AAA tissues, whereas γ-secretase inhibitor DBZ markedly attenuated these effects (Figure 4). Taken together, the results demonstrate that activation of *Notch*1 increases AAA formation partly by inducing M1 versus M2 polarization of macrophages.

*Notch* receptors are known to be expressed in developing and mature lymphocytes and in lymphoid tissues, suggesting a role in both lymphocyte development and peripheral maturation, especially Th cell polarization [31]. Upon activation, CD4^+^ T lymphocytes have the capacity to differentiate into several distinct subsets including the Th1 and Th2 phenotypes [8,18]. The alteration of Th1 (IFN-γ) and Th2 (IL-4 and IL-10) cytokines contributes to M1/2 phenotypic change of macrophages [16,29]. Recent studies reported that *Notch*1 inhibition reduced infiltration of CD3^+^ T lymphocytes at the site of aneurysm tissues, but no effect of *Notch* on CD4^+^ or CD8^+^ T cell differentiation were observed in the aneurysmal aorta or spleen between Apo E^−/−^ and Notch1^+/−^ApoE^-/-^groups after Ang II treatment [3,28]. However, several other studies have demonstrated that Th2 cytokines and responses play an important role in AAA formation [29,31-33]. For example, Th2 cytokines such as IL-4, IL-5, and IL-10 are highly expressed in AAA tissues compared with nondiseased tissue or stenotic atheroma. In contrast, AAAs had low levels of Th1 cytokines including IL-2 and IL-15 [29,33]. Meanwhile, IFN-γ is capable of attenuating AAA lesions [29]. Blockade of IFN-γ action results in IL-4 driven inflammation, directly increases expression of MMP-9 and -12 leading to aneurysm formation [29]. Moreover, several studies have demonstrated that *Notch* pathway blockade has profound implications, suppressing vessel wall inflammation, cytokine production, and T cell accumulation [8,10,30]. GSIs treatment reduces allergic pulmonary inflammation by decreasing Th2 cytokine production and concomitantly increasing Th1 cytokine secretion [10]. There is growing evidence that activation of the *Notch*1 signaling pathway regulates T cell differentiation and the expression of an inflammatory cascade [10]. Consistent with these results [29,31-33], our findings indicated that Ang II infusion significantly increased the proportions of total CD3^+^ and CD4^+^ T cells in the serum or the AAA tissue, and increased the expression of Th2 cytokines IL-4 and IL-10 in the AAA tissues. Moreover, Ang II decreased T-bet/GATA-3 expression ratios, which enhanced induction of the Th2 response. However, DBZ treatment markedly attenuated these effects (Figure 5). Collectively, activation of *Notch*1 signaling mediates AAA formation at least partially by promoting CD4^+^ T cells to differentiate into Th2 phenotype. 

Neovascularization is a critical event in aneurysm formation. Data have suggested that Ang II-induced development and rupture of AAA are associated with increased neovascularization of the media and adventitia [25,34]. Several studies demonstrated that *Notch* signaling played an important role in angiogenesis. A recent study suggested that *Notch* activation mediated VEGF-induced dermal angiogenesis in vivo. Treatment with ERK ihibitor attenuates angiogenesis and Ang II-induced AAA formation in Apo E^-/-^ mice [34]. Consistent with these results, our data demonstrated that Ang II stimulated a dramatic increase in neovascularization, the mRNA expression of VEGF and HIF, and the levels of ERK and AKT phosphorylation in the aortas of AAA in Apo E^-/-^ mice, whereas these effects Ang II on the expression of VEGF and HIF, and the levels of ERK phosphorylation were markedly decreased in the aortas of Apo E^-/-^ mice treated with DBZ. Thus, these data suggest that *Notch* signaling takes part in the angiogenesis to partially promote aneurysm formation; otherwise, DBZ can reduce it by inhibiting the *Notch* signaling-mediated actions. 

 Though we have performed experiments to test the effects of Notch inhibition by γ-secretase inhibitor on the accumulation of inflammatory cells, expression of MMP and angiogenesis, which are known to play critical roles in AAA formation, there are still some limitations of the study (1). Studies are needed to further prove the influence of Notch inhibition on smooth muscle cell (SMC) apoptosis, proliferation and differentiation, which are required for the development of AAA (2). Notch1 inhibitors such as γ-secretase are potential therapeutic agents for treatment of tumor, Alzheimer’s disease and cardiovascular diseases [6,7,9,10]. The present study and other also demonstrated the beneficial effects of pharmacological inhibition of Notch signaling on Ang II-induced AAA formation. Further investigations in other animal models of AAA are needed to determine the clinical use of Notch inhibitor DBZ as a pharmacological therapy.

In conclusion, our data demonstrate that *Notch*1 signaling pathway plays a critical role in AAA formation. This was associated with accumulation of macrophages and CD4^+^ T cells, Th2 differentiation and ERK-mediated angiogenesis in Ang II-induced AAA tissues. In contrast, the *Notch* γ-secretase inhibitor DBZ markedly inhibited these effects. Thus, these results suggest that activation of *Notch*1 signaling promotes the development of AAA by multiple mechanisms, and the γ-secretase inhibitor DBZ might be a new therapeutic target for treating AAA formation.

## Materials and Methods

All animal protocols were approved by the Animal Care and Use Committee of Capital Medical University (20120109) and experiments conformed to the Guide for the Care and Use of Laboratory Animals (National Institutes of Health publication No.85-23,1996).

### Antibodies and reagents

Antibodies to IL-6, *Notch* ICD, alpha-smooth muscle actin (α-SMA) and pCNA were purchased from Abcam Inc.; antibodies to MMP-2, MMP-9, MCP-1 and Mac-2 were obtained from Santa Cruz Biotechnology; antibodies to CD3 were from Bioworld, to CD4 were from Novus, to CD8 (catalog number:AJ-1170c) was from Abgent, to Isolectin (catalog number: FL-1201) were from Vector Laboratories, to TUNEL were from Merck, to HES-1 were from Millipore Biosciences; antibodies against phospho-ERK1/2, ERK1/2, phospho-AKT, AKT, β-actin, and anti-mouse or anti-rabbit conjugated antibodies were purchased from Cell Signaling Technology. For Flow Cytometry, antibodies to CD3, CD4, CD8 and CD11b were from BD Bioscience Pharmingen; antibody to CD206 was from Biolegend. γ-secretase inhibitor dibenzazepine (DBZ) was purchased from Santa Cruz Biotechnology. Ang II was from Sigma. Penicillin, streptomycin, and fetal bovine serum (FBS) were obtained from Invitrogen Life Technologies. 

### Tissue sample processing

Abdominal aortic tissues and blood were obtained from patients with AAAs and control patients without AAA who underwent surgical procedures. Abdominal aortic aneurysms were confirmed by aortic morphological analysis as described previously [4]. The control subjects were obtained from heart transplant donors, who have similar clinical characteristics, including age, sex, ethnic background, etc. The study protocol was approved by the Ethical Committee of Peking Union Medical College Hospital, and informed consent was obtained from the individuals.

### Animals and treatment

Male wild-type (WT) C57BL/6J and Apo E^–/–^ mice were obtained from were purchased from the Jackson Laboratory (Bar Harbor, Me). All mice were bred as littermate controls, and housed in a pathogen-free barrier facility, and were fed a normal laboratory diet throughout experimentation. Mice at 10-12 weeks of age were infused subcutaneously for 4 weeks with saline vehicle or Ang II at a dose of 1000 ng.kg^-1^.min^-1^ (Sigma-Aldrich, St. Louis, MO) with Alzet osmotic minipumps (Model 2004, Durect Corporation) as described previously [1]. Ang II-treated mice were received an intraperitoneal injection of either saline vehicle or γ-secretase inhibitor, dibenzazepine (DBZ) (1 mg/kg/d, dissolved in saline, Santa Cruz Biotechnology) 1 day before mini-pump implantation, and the treatment continued daily for 4 weeks as described [4]. The blood pressure was measured in conscious mice using a computerized tail-cuff system (Softron BP-98A; Softron, Tokyo, Japan) as described [35,36]. All mice were anesthetized. The aortic tissues were removed and prepared for further histological and molecular analysis. 

### Analysis and quantification of AAA

Mice were anesthetized with 1.5% isoflurane by inhalation. Abdominal aortic diameter was monitored with a Vevo 770 ultrasound system (VisualSonics Inc.) and MNI, respectively, as described [36]. After perfusion with 4% paraformaldehyde, the aorta was exposed under a dissecting microscope, and the periadventitial tissue carefully removed from the aortic wall. The out diameter of the suprarenal aorta was also measured with a caliper. For quantifying aneurysm incidence, an aneurysm was defined as a 50% or greater increase in the external width of the suprarenal aorta compared to aortas from saline-infused mice as described previously [4]. Aneurysm severity was rated from Type I to Type IV according to the method of Daugherty et al [13].

### Histology, immunohistochemical staining and TUNEL assay

The mice were killed after 4 weeks of treatment. For morphological analyses, aortas were perfused with normal saline and fixed with 10% PBS and formalin for 5 min. Whole aortas were harvested and aortic tissue was removed from the ascending aorta to the ileac bifurcation. The tissue was laid out on a black background, and an image of the aorta was recorded. After fixed for 24 h and embedded in paraffin. Cross-sections (5 μm) were prepared. Paraffin sections were stained with H&E, Masson as described [36,38]. Immunohistochemical staining was performed with primary antibodies, including Mac-2, MCP-1, IL-6, NICD, CD3, CD4, PECAM, α-SMA, MMP-2, MMP-9 and Hes1. TUNEL assay was described as previously [4,12]. Counterstaining was performed with DAPI (Santa Cruz Biotechnologies) for 10 min at room temperature. Digital photographs were taken at 10X or 20X magnification of over 10 random fields from each aorta, and the positive areas were calculated by software (NIKON).

### Flow cytometry

Peripheral blood was collected into EDTA (1.8 mg/ml) at the termination and mononuclear cells were isolated via lysis of red blood cells with an ammonium chloride solution. Peripheral blood populations were determined via flow cytometry analysis as described [37,38].

### Quantitative real-time PCR analysis

Total RNA was isolated by Trizol reagent (Invitrogen Corp.). For real-time RT-PCR, cDNA was synthesized from 500 ng of total RNA using TaqMan Gold RT-PCR Kit (Applied Biosystems) according to the manufacturer’s protocol. Quantitative real-time PCR (qPCR) was performed with an iCycler IQ system (Bio-Rad) as described [35,38]. The cDNA samples were diluted 20-fold, and real-time PCR reaction was carried out using SYBR green JumpStart Taq ReadyMix (Sigma-Aldrich) with 100 μM of primer. Amplifications were performed in an ABI PRISM 7000 Sequence Detection System (Applied Biosystems). Thermal cycler conditions were 50°C for 2 minutes and 95°C for 10 minutes to activate/inactivate different enzymes, then 40 cycles of 15 seconds at 95°C followed by 1 minute at 59°C (annealing and extension). All samples were normalized to the relative levels of GAPDH and results expressed as fold increase in relative levels. Primers were designed using PrimerExpress (Applied Biosystems) software as follows (Table 1).

**Table 1:** Primers for real-time PCR analysis.

Genes	Forward primers	Reverse primers
IFN-γ	5'-TCTGGAGGAACTGGCAAAAG-3'	5'-TTCAAGACTTCAAAGAGTCTGAGG-3'
TNF-α	5'-GGCAGGTCTACTTTGGAGTCATTG-3'	5'- GTTAGAAGGACACAGACTGG-3'
IL-4	5'-TCAACCCCCAGCTAGTTGTC-3'	5'-TGTTCTTCGTTGCTGTGAGG-3'
IL-6	5'-AGTTGCCTTCTTGGGACTGA-3'	5'-TGGGTGGTATCCTCTGTGAAG-3'
IL-10	5'-AGTTGCCTTCTTGGGACTGA-3'	5'-TGGGTGGTATCCTCTGTGAAG-3'
MMP-2	5'-ACACTGGGACCTGTCACTCC-3'	5'-TGTCACTGTCCGCCAAATAA-3'
MMP-9	5'-CAATCCTTGCAATGTGGATG-3'	5'-AGTAAGGAAGGGGCCCTGTA-3'
TIMP-1	5′-GCAACTCGGACCTGGTCATAA-3′	5′-CGGCCCGTGATGAGAAACT-3′
VEGF	5'-CCTGGTGGACATCTTCCAGGAGTACC-3'	5'-GAAGCTCATCTCTCCTATGTGCTGGC-3'
HIF-1α	5'-CCCAATGGATGATGATTTCC-3'	5'-TGGGTAGAAGATGGAGATGC-3'
GATA	5′-AGAACCGGCCCCTTATCAA-3′	5′-AGTTCGCGCAGGATGTCC-3′
T-bet	5′-CAACAACCCCTTTGCCAAAG-3′	5′-TCCCCCAAGCAGTTGACAGT-3′
GAPDH	5'-TGTACCGTCTAGCATATCTCCGAC-3'	5'-ATGATGTGCTCTAGCTCTGGGTG-3'

### Statistical analysis

All data were analyzed using GraphPad software (GraphPad Prism version 4.00 for Windows; GraphPad Software). Results are expressed as mean ± SEM. Differences were analyzed by Student's unpaired *t* test or ANOVA followed by the Newman–Keuls multiple-comparison test. A value of *P* < 0.05 was considered to be statistically significant.
